# The effect of the SAFE intervention on post-discharge suicidal behavior: a quasi-experimental study using propensity score matching

**DOI:** 10.1007/s00127-023-02585-y

**Published:** 2023-11-23

**Authors:** Trine Madsen, Annette Erlangsen, Eybjørg Egilsdottir, Per Kragh Andersen, Merete Nordentoft

**Affiliations:** 1 Danish Research Institute for Suicide Prevention, CORE- Copenhagen Research Center for Mental Health, Mental Health Centre Copenhagen, Copenhagen, Denmark; 2 Section of Epidemiology, Faculty of Health and Medical Sciences, University of Copenhagen, Copenhagen, Denmark; 3 Department of Mental Health, Johns Hopkins Bloomberg School of Public Health, Baltimore, MD USA; 4 Center of Mental Health Research, Australian National University, Canberra, Australia; 5 Section of Biostatistics, Faculty of Health and Medical Sciences, University of Copenhagen, Copenhagen, Denmark; 6 Department of Clinical Medicine, Faculty of Health Science, University of Copenhagen, Copenhagen, Denmark

**Keywords:** Suicide, Mental health, Hospitalization, Epidemiology

## Abstract

**Objectives:**

The risk of suicidal behavior after discharge from psychiatric admission is high. The aim of this study was to examine whether the SAFE intervention, an implementation of a systematic safer discharge procedure, was associated with a reduction in suicidal behavior after discharge.

**Methods:**

The SAFE intervention was implemented at Mental Health Center Copenhagen in March 2018 and consisted of three systematic discharge procedures: (1) A face-to-face meeting between patient and outpatient staff prior to discharge, (2) A face-to-face meeting within the first week after discharge, and (3) Involvement of relatives. Risk of suicide attempt at six-month post-discharge among patients discharged from the SAFE intervention was compared with patients discharged from comparison mental health centers using propensity score matching.

**Results:**

7604 discharges took place at the intervention site, which were 1:1 matched with discharges from comparison sites. During the six months of follow-up, a total of 570 suicide attempts and 25 suicides occurred. The rate of suicide attempt was 11,652 per 100,000 person-years at the SAFE site, while it was 10,530 at comparisons sites. No observable difference in suicide attempt 1.10 (95% CI: 0.89–1.35) or death by suicide (OR = 1.27; 95% CI:0.58–2.81) was found between sites at 6-month follow-up.

**Conclusion:**

No difference in suicidal behavior between the sites was found in this pragmatic study. High rates of suicidal behavior were found during the 6-months discharge period, which could suggest that a preventive intervention should include support over a longer post-discharge period than the one-week follow-up offered in the SAFE intervention.

**Supplementary Information:**

The online version contains supplementary material available at 10.1007/s00127-023-02585-y.

## Introduction

It is well established that the period shortly after discharge from psychiatric inpatient facilities is associated with a high risk of suicide [1–5]. Regardless of psychiatric diagnoses, the suicide rate during first week after discharge is more than 100-fold higher than the rate for individuals who have not been admitted to psychiatric hospital [3]. Thus, as part of standard care at psychiatric wards in Denmark, a suicide risk assessment is generally conducted at the time of discharge to determine whether it is safe to send the patient home. Although most people are considered not at acute risk of suicide at the time of discharge [6], it may be challenging to account for non-clinical factors, such as the home environment and current living conditions of the patient, when arriving home [7]. Many people who are hospitalized for psychiatric disorders in Denmark face challenging situations, as 18% are without a job, 23% on disability pensions and 16% have previously received treatment for alcohol- or substance misuse [8]. It is, therefore, not unlikely that patients may encounter stressful and chaotic situations when being discharged, which potentially could trigger suicidal thoughts and behaviors. These may be further exacerbated through low support from the social network. The Danish Health Data Authority reports that the average number days per psychiatric admission has declined from 24.3 days per admission in 2010 to 19.2 in 2017. Shorter admission times could imply a higher pressure for discharging too soon to make room for new patients; consequently, some patients may not be considered fully recovered at the time of discharge, thus, emphasizing the importance of attendance in continued care in outpatient settings.

In meta-analyses, brief suicide interventions offered during high-risk periods, such as time of discharge, have been linked to a 30–40% reductions in suicidal behavior (i.e., suicide attempt and suicide) [9, 10]. Thus, a safety plan consisting of strategies on how to manage crisis, for instance, trying to distract oneself from suicide thoughts and removing access to lethal objects, such as medicine or guns has been suggested as a promising tool for people at risk of suicide [9, 10]. In addition, other brief interventions, such as follow-up contact after discharge (by telephone, mail, text, postcard) and care coordination (planning follow-up contact with psychiatric services) have been associated with reductions. Most of these follow-up interventions were not provided face-to-face, but instead over the phone or in a text format. A face-to-face meeting between the patient and the healthcare provider from the outpatient clinic prior to discharge was linked to a higher compliance with continuation of treatment after discharge [11–13], which is a promising feature because discontinuation of treatment has been linked to suicidal behavior [14, 15]. For this reason, it is plausible that a face-to-face meeting might prevent suicidal behavior. Ideally, a such meeting should be conducted in in the patient’s home, as this may give the health care provider an opportunity to assess the patient’s recovery and social situation after discharge, in addition, to reducing likelihood of a ‘no show’. The SAFE intervention consisted of a face-to-face meeting in first week after discharge and was designed to ensure a continued care during the transitioning from being admitted to living at home. The goal of the SAFE intervention was to identify and prevent suicidal behavior during the critical post-discharge phase.

The aim of this study was to examine whether patients who were discharged from the SAFE-intervention site had lower risks of suicide attempt and suicide than a propensity-matched comparison group of patients discharged from control sites when assessed six months after discharge.

## Methods

### Study design, setting, and data sources

We applied a quasi-experimental design where individuals discharged from one mental health center (MHC) were offered the SAFE intervention and compared to a propensity-score-matched sample of individuals discharged from other MHCs in the same region. The study may be considered as a natural experiment because individuals were included into the study if they lived in the catchment areas of the studied MHCs. The catchment area consisted of eight MHCs within the Capital Region of Denmark. Each center served as a defined catchment area. The SAFE intervention was implemented at MHC Copenhagen, while four adjacent MHCs, MHC Ballerup, MHC Glostrup, MHC Amager, and MHC North Zealand, were used for the comparison. The three other MHCs in the Capital Region of Denmark were excluded from the study; MHC Bornholm, which was situated on an island two hours from Copenhagen city with a different population and level of urbanicity; Psychotherapeutic Center Stolpegård, which exclusively provided outpatient treatment; and MHC Sct. Hans, which was a specialized center for treatment of forensic patients and patients with dual diagnosis. The SAFE project was implemented from March 1, 2018 to March 31, 2020. Information on individuals discharged from the included psychiatric hospitals was obtained from national hospital registers, in which all in-, out-, and emergency department patients seen at psychiatric hospitals in Denmark since 1995, have been recorded. Individual-level data on discharges prior to 2. February 2019 were obtained from the Danish Psychiatric Research Register [16]. After this date, data on all discharges were derived from the National Patient Register. Each hospital in Denmark has an id number, and this id number was used to identify inpatients discharged from each of the five MHCs [17]. Psychiatric diagnoses were classified according to the International Classification of Diseases, version 10 (ICD-10) [18].

### Participants

We obtained data on all patients, aged 18 or above, discharged from one of the five MHCs. Each discharge was considered as a separate unit of follow-up, i.e., if a patient was admitted to psychiatric hospital several times during the follow-up, each discharge would be analyzed separately in the study. Patients were excluded if they had been recorded with primary diagnoses of one of the following disorders: organic psychiatric disorders (ICD-10: F00–F09), eating disorders (ICD-10: F50–F59), mental retardation (ICD-10: F70–F79), pervasive and specific developmental disorders (ICD-10: F80–F89), and behavioral and emotional disorders with onset usually occurring in childhood and adolescence (ICD-10: F90–F98). Individuals with these disorders (*n* = 361) were excluded because they attended specialized outpatient treatment.

### Intervention

The SAFE intervention consisted of the following three components, which were systematically implemented: (1) Face-to-face introduction to SAFE prior discharge where eligible inpatients at the intervention site were invited to participate in the project during a personal meeting with a health care provider from the outpatient clinic, while still being in admission. During this first meeting, the health care provider from the outpatient clinic interviewed the patient regarding their home environment and together they complied a safety plan as well as scheduled a face-to-face meeting for first week after discharge. The health care providers professional backgrounds were social workers, nurses, occupational therapist or psychologists.; (2) A face-to-face meeting during the first week after discharge was held between patient and, ideally, the same health care provider, which they met at the hospital ward. The meeting was preferably conducted as a home visit, but patients could also request to meet in different venue, for instance, the outpatient clinic or at café. The home visit allowed the clinician to assess the patients’ post-discharge situation, i.e., identify possible stressors, monitor the plans for follow-up, review the safety plan, and reassess the suicide risk as well as inform the patient about suicide warning signs; and (3) Involvement of relatives where patients were encouraged to involve relatives in their treatment course as relatives can provide emotional and practical support to the patient in a vulnerable situation. If involved, the health care provider would inform the relatives on suicide warning signs, as this might foster faster help-seeking if the patient’s condition should deteriorate.

The SAFE intervention was compared with treatment-as-usual offered at the comparison sites, which depending on severity of condition and disorder consisted of reference to a variety of services. These services could be referral to a private psychiatrist, substance abuse treatment, psychotherapy or follow-up care in secondary health care and the availability of appointments to these services are often several weeks due to waitlists. Also, some were referred at discharge to contact their general practitioner when at home. The overall difference to the intervention was that in SAFE, everyone, no matter of condition or disorder, was systematically offered to receive a face-to-face follow-up in the week after discharge where the risk of suicide is really high whereas in treatment-as-usual many did not have a follow-up due to waitlist problems.

### Outcomes

The outcomes of interest were suicide and suicide attempt, respectively. Suicide deaths were identified in the Cause of Death Register as one of the following ICD-10 codes: X60–X84 or Y87.0 [19].

Suicide attempt was defined as a presentation to either psychiatric or somatic hospitals, including emergency departments where a suicide attempt had been recorded using the following ICD-10 codes X60–X84 or when reason for contact was indicated as suicide attempt in both The National Patient Register and The Danish Psychiatric Research Register. In addition, the following combinations of ICD diagnoses were included as hospital contacts due to suicide attempt: a main diagnosis of a mental disorder (ICD-10: F00–F99) together with one of the following sub-diagnoses: S51, S55, S59, S61, S65, S69 (cutting by sharp objects), T36–T50 (poisoning by pharmaceuticals), T52–T60 (poisoning by non-pharmaceuticals) as well as all admissions with a main diagnosis of T39, T40 (poisoning by mild analgesics; except T40.1), T42, T43, and T58 (poisoning by opioids, psychotropics, and by carbon monoxide).

### Follow-up

Participants were followed from date of discharge until episode onset of the studied outcome, new psychiatric admission, death, emigration or six months post-discharge, which ever occurred first.

### Power calculation

Based on estimates on previous population attributable risk calculations [20], approximately 40 suicidal acts in the first week after discharge and approximately 200 in the first 6 months after discharge were expected. With approximately 6000 discharges, our power calculation (based on a statistical significance level of 5 percent and a power of 90 percent) shows that we have the power to find a difference in suicidal acts of 7% versus 5% between the control and SAFE sites, corresponding to a 28 percent reduction in suicidal behavior.

### Statistical analysis

Individuals discharged from the SAFE intervention site were matched to individuals from the comparison group using exact matching and a propensity score. The unit for the matching was discharges not individuals, hence, if an individual was discharged more than once from the intervention site then a matching was conducted for each discharge. The propensity score was derived from following socio-demographics and health-related variables from nationwide registers: sex (male, female); age group (18–29 years, 30–44 years, 45–64 years, 65 + years); socio-economic status (working, unemployed/social or sickness benefit, retired, missing); highest achieved educational level (elementary school, vocational training, high school, bachelor degree or higher, missing); civil status (married, divorced, never married, missing or other); placed in foster care; primary diagnoses of substance misuse (ICD-10: F10–F19), schizophrenia spectrum disorders (ICD-10: F20–F29), affective disorders (ICD-10: F30–F39), anxiety or stress-related disorders (ICD-10: F40–F49) or personality disorders (ICD-10: F60–F69); admission lasted more than 7 days; ever diagnosed with substance misuse; ever diagnosed with schizophrenia spectrum diagnosis; ever diagnosed with affective disorders; previous suicide attempt; prescribed antipsychotic medication within 12 months of being discharged; prescribed antidepressant medication within 12 months of being discharged; parents with history of psychiatric disorder; and parents with history of a suicide attempt or suicide. Date of discharge was used as time point for the matching and all matching factors were measured on this date. Matching factors were selected because they were considered as predictors of suicidal behavior or factors, which might have described differences between the intervention and comparison groups. An exact matching was prioritized for previous suicide attempt and prescribed antidepressant medication within 12 months of being discharged and a propensity score was calculated for the remaining variables. After having identified a matched comparison group with a 1:1 ratio, odds ratios (OR) of suicide and suicide attempt were calculated in separate models with their 95% confidence intervals at 2 weeks, 1 months and 6 months after discharge. We accounted for multiple discharges of the same individual through adjustments for repeated subjects in the statistical models. Following sensitivity analyses were conducted: Firstly, the first months of the SAFE intervention (from March 1, 2018 to August 31, 2018) were omitted, implying that only discharges after September 1, 2018 onwards were included, to ensure a higher level of adherence to the SAFE intervention, as it might have taken time to implement new procedures into clinical practice. Second, we excluded all patients discharged with a main diagnosis of depression or bipolar disorder, as members of this group were enrolled in a congruent research project during half of the follow-up and, thus, precluded from participating in the SAFE intervention. Third, individuals were censored after a first recorded suicide attempt to test that the risk estimate for suicide attempt in the primary analyses would not be biased by individuals with multiple suicide attempts.

All analyses were conducted using SAS version 9.4 and the cut-off for statistical significance level was set at *p* = 0.05.

## Results

In all, 30,031 discharges of 13,958 individuals occurred between March 1, 2018 and March 31, 2020 at the five MHCs in the Capital Region of Denmark. Of these, 7604 (25%) discharges of 3740 (27%) individuals were from MHC Copenhagen, where the SAFE intervention was implemented, while the remaining 22,427 (75%) discharges of 10,218 (73%) individuals occurred at the four other MHCs (Table 1).

**Table 1:** Distribution of propensity matching factors for discharges from SAFE the intervention site and the comparison sites

	Discharges: SAFE intervention site (*n*=7604)		Unmatched discharges: comparison sites (*n*=22427)		Diff. in % to SAFE	Matched discharges: comparison sites (*n*=7604)		Diff. in % to SAFE
Variables	*n*	%	*n*	%		*n*	%	
Sex								
Female	3605	47.4	10560	47.1	0.3	3588	47.2	0.2
Male	3999	52.6	11867	52.9	− 0.3	4016	52.8	− 0.2
Agegroups								
18–29 years	2079	27.3	6185	27.6	− 0.2	2166	28.5	− 1.1
30–44 years	2333	30.7	6052	27.0	3.7	2222	29.2	1.5
45–64 years	2437	32.0	7670	34.2	− 2.2	2438	32.1	0.0
65+ years	755	9.9	2520	11.2	− 1.3	778	10.2	− 0.3
Socio-economic status								
Working	1862	24.5	5185	23.1	1.4	2001	26.3	− 1.8
Unemployed, social or sickness benefit	4402	57.9	13012	58.0	− 0.1	4243	55.8	2.1
Retired	1288	16.9	4129	18.4	− 1.5	1309	17.2	− 0.3
Missing	52	1.0	101	0.0	1.0	51	1.0	0.0
Highest educational status								
Elementary school	3025	40.0	10680	48.0	− 8.0	3179	42.0	− 2.0
Vocational training	1525	20.1	5243	23.4	− 3.3	1562	20.5	− 0.5
High school	1108	14.6	2044	9.1	5.5	1153	15.2	− 0.6
Bachelor degree or higher	1424	18.7	3061	13.6	5.1	1288	16.9	1.8
Missing	522	7.0	1399	6.0	1.0	422	6.0	1.0
Civil status								
Married	731	9.6	3050	13.6	− 4.0	733	9.6	0.0
Divorced	1294	17.0	4393	19.6	− 2.6	1254	16.5	0.5
Never married	5413	71.2	14286	63.7	7.5	5441	71.6	− 0.4
Missing or other	166	2.0	698	3.0	− 1.0	176	2.0	0.0
Placed in foster care								
Yes	1265	16.6	3899	17.4	− 0.7	1215	16.0	0.7
Primary diagnosis								
Substance misuse	1239	16.3	4088	18.2	− 1.9	1220	16.0	0.2
Schizophrenia spectrum disorder	3230	42.5	9639	43.0	− 0.5	3158	41.5	0.9
Affective disorders	1594	21.0	3672	16.4	4.6	1657	21.8	− 0.8
Anxiety or stress related disorder	732	9.6	2543	11.3	− 1.7	792	10.4	− 0.8
Personality disorders	397	5.2	1145	5.1	0.1	343	4.5	0.7
Admitted more than a week								
Yes	3563	46.9	9315	41.5	5.3	3686	48.5	− 1.6
History of psychiatric comorbidity								
Substance misuse diag	4523	59.5	13025	58.1	1.4	4518	59.4	0.1
Schizophrenia spectrum disorder	4992	65.6	14536	64.8	0.8	4901	64.5	1.2
Affective disorders	4319	56.8	12436	55.5	1.3	4322	56.8	0.0
Previous suicide attempt								
Yes	2691	35.4	8535	38.1	− 2.7	2691	35.4	0.0
Prescriptions last 12 months								
Anti-psychotic medicine (yes)	6069	79.8	18612	83.0	− 3.2	6068	79.8	0.0
Anti-depressive medicine (yes)	5131	67.5	16045	71.5	− 4.1	5131	67.5	0.0
Parent with history of psychiatric disorder								
Yes	1982	26.1	5750	25.6	0.4	1872	24.6	1.4
Parent with history of suicidal behavour								
Yes	622	8.2	2584	11.5	− 3.3	537	7.1	1.1

When matching discharges from the SAFE intervention site with discharges from the comparison sites using the propensity score, the range of the percentual differences across the matched factors was reduced from 0.1%–8.0% to 0.0%–2.1%. After the 1:1 matching, the sample consisted of 15,208 discharges, which were followed for up to six months. During follow-up, 570 suicide attempts were recorded. Of these, 298 suicide attempts were by patients from the SAFE intervention site; corresponding to a rate of 11,652 (95% CI: 10,329–12,975) per 100,000 person-years (Table 2). Among the matched comparisons, a total of 272 suicide attempts were observed, resulting in a rate of 10,530 (95% CI: 9279–11,782) per 100,000 person-years. Out of 25 suicide deaths, 14 occurred among patients from the SAFE intervention site, corresponding to a rate of 538 (95% CI: 256–820) per 100,000 person-years, whereas 11 suicides and a rate of 420 (95% CI: 172–668) was found among comparisons over the 6-month follow-up.

**Table 2:** Frequencies by intervention group and odds ratios (95% confidence intervals) for suicide attempt and suicide in SAFE versus comparison sites after 2-week, 1-month, and 6-month follow-up

	Events SAFE site (*N* = 7604)	Events comparison sites (*N* = 7604)	Odds ratio (95% CI)	*P* value
Suicide attempt after				
Up to 2 weeks	101	82	1.23 (0.89–1.71)	0.20
Up to 1 month	151	122	1.24 (0.93–1.65)	0.13
Up to 6 months	298	272	1.10 (0.89–1.35)	0.37
Suicides after				
Up to 2 weeks	3	4	0.75 (0.17–3.35)	0.71
Up to 1 month	6	5	1.20 (0.37–3.93)	0.76
Up to 6 months	14	11	1.27 (0.58–2.81)	0.55

We found no statistically significant difference between individuals discharged from the intervention site and the comparison sites with respect to suicide attempt at 2 weeks of follow-up (OR = 1.23; 95% CI: 0.89–1.71). The same applied after 1 month (OR = 1.24; 95% CI: 0.93–1.65) and 6 months of follow-up (OR = 1.10; 95% CI: 0.89–1.35). There was also no observable difference with respect to death by suicide between individuals discharged from the SAFE intervention site and their matched comparisons with an OR of 1.27 (95% CI:0.58–2.81) at 6 months of follow-up. None of sensitivity analyses revealed significant differences in outcomes between those discharged from the intervention and comparison sites (Table 3 and eTable 1).

**Table 3:** Sensitivity analyses—Frequencies by intervention group and odds ratios (95% confidence intervals) for suicide attempt and suicide in SAFE versus comparisons sites, respectively, after 2-week, 1-month, and 6-month follow-up

Sensitivity analysis 1	Study period September 1 2018–March 31 2020			
	Events SAFE site (*N* = 5857)	Events comparison sites (*N* = 5857)	Odds ratio (95% CI)	*P* value
Suicide attempts after				
up to 2 weeks	74	59	1.26 (0.87–1.82)	0.22
up to 1 month	107	83	1.29 (0.93–1.80)	0.13
up to 6 months	219	196	1.12 (0.89–1.41)	0.33
Suicides after				
up to 2 weeks	na^1^	na^1^	0.33 (0.07–1.65)	0.18
up to 1 month	4	8	0.50 (0.15–1.66)	0.26
up to 6 months	12	14	0.86 (0.40–1.85)	0.69

Sensitivity analysis 2	Excluding all discharges where patient had a primary diagnosis of unipolar or bipolar disorder			
	Events SAFE site (*N* = 6010)	Events comparison sites (*N* = 6010)	Odds ratio (95% CI)	*P* value
Suicide attempts after				
up to 2 weeks	79	68	1.16 (0.81–1.68)	0.42
up to 1 month	115	96	1.20 (0.86–1.68)	0.28
up to 6 months	231	188	1.24 (0.97–1.57)	0.08
Suicides after				
up to 2 weeks	na^2^	na^2^	1.00 (0.20–4.96)	1.00
up to 1 month	5	6	0.83 (0.25–2.73)	0.76
up to 6 months	12	11	1.09 (0.48–2.47)	0.83

Sensitivity analysis 3	Follow-up until first suicide attempt			
	Events SAFE site (*N* = 7071)	Events comparison sites (*N* = 7071)	Odds ratio (95% CI)	*P* value
Suicide attempts after				
up to 2 weeks	60	62	0.97 (0.68–1.38)	0.86
up to 1 month	96	88	1.09 (0.82–1.46)	0.55
up to 6 months	201	207	0.97 (0.80–1.18)	0.76

na^1^: Too few observations to report by intervention group—8 individuals died by suicide within 2-week period

na^2^: Too few observations to report by intervention group—6 individuals died by suicide within 2-week period

## Discussion

High rates of both suicide attempt (> 10,000 per 100,000 person-years) and suicide (> 400 per 100,000 person-years) were found among patients discharged from psychiatric admission during the follow-up of this brief intervention study. However, our results indicated no observable differences in these rates when comparing patients discharged from the intervention site with those discharged from the comparison sites.

Conflicting effects have been reported for brief suicide interventions targeting critical high-risk periods. While some interventions resulted in statistically significantly reduced risk of suicide attempt [21–26], others did not find this [27–30]. Most of the previously conducted interventions included same elements as examined here, such as interview before discharge with a focus on suicide warning signs and safety planning as well as follow-up contact after discharge (either by phone or post cards). Except for two interventions [23, 24], most have been conducted as randomized controlled trials (RCT) [21, 22, 25–31]. All RCTs had small sample sizes and, consequently, only few events of suicide attempt were observed during follow-up; implying that they might not have been sufficiently powered to detect a statistical difference. The two studies, which did not include a randomized allocation, were cohort studies of larger samples (*n* = 1376[23] and *n* = 1640[24]) and both identified an effect of the brief intervention over treatment-as-usual (TAU). This is further supported by the findings from two meta-analyses [9, 10] in which these studies were both included. Our study sample was substantially larger (*n* = 15,208) and we, consequently, observed a large number of events. Still, we did not find that the SAFE intervention was linked to a reduction in risks of suicide attempt and suicide. One reason for this might be differences in study populations; the SAFE intervention was offered to all discharged inpatients whether they had suicidal tendencies or not, while participants in the other studies either experienced current suicidal ideations or had recently presented with a suicide attempt.[21, 22, 24–26]. Targeting all patients, as we did may dilute the effectiveness of the intervention, however we choose to offer the intervention to all discharged patients because we know from previous studies that only focusing on those who already had displayed suicidality, we risk bypassing many who will be at suicidal risk after discharge [32]. It is also possible that the SAFE intervention, as a consequence of the pragmatic design, was not offered as stringently as in RCT studies. Due to a new registration system, which was implemented at the MHCs during the follow-up, it was not feasible to register who and how many individuals were offered and/or consented to participate in the SAFE intervention. In other words, we could not assess the fidelity of the study. Still, an internal quality assessment, based on journal audits, suggested a high compliance. During the first months of the SAFE intervention, i.e., March–October 2018, the number of discharged patients who received a face-to-face meeting within 7 days rose from 28 to 77%. Also, the number of recorded suicide risk screenings conducted during the first week after discharge rose from 3 to 19%. During the follow-up, outpatient clinics at control sites initiated competitive efforts, in form of the Flexible Assertive Community Teams (F-ACT) [33] and Acute Teams. These efforts consisted of community outreach for individuals with severe mental illnesses, provided through clinical outpatient teams and, which were offered in the critical post-discharge phase. This may have biased our evaluation as these efforts could be viewed as comparable to the one of the SAFE procedures, namely home-visits during first week after discharge.

Irrespective of a significant effect, there remains a strong rationale for providing the three procedures of the SAFE intervention in clinical practice. Recently, F-ACT have proved to increase the number of outreach visits after discharge as well as outpatient contact with relatives of patients [33], which as mentioned in the introduction may be linked to lower suicide risk. Based on existing evidence, it is possible that these initiatives are most effective when offered to patients who recently suffered from suicidal ideations or behaviors as opposed to offering to all discharged patients, although only focusing on those who presented with suicide attempt/severe suicidal thoughts we risk bypassing offering follow-up help to individuals who become suicidal after discharge.

### Strengths and limitation

Besides not being designed as an RCT, not having individual-level information on who received the SAFE intervention, and a lack of fidelity measures, other limitations include (1) the fact that suicide attempts may be underreported as only hospital-recorded incidents were captured, (2) ascertainment bias if those discharged from the SAFE intervention site due to higher expose of safety planning may increase the likelihood that that these individuals sought hospital care for suicide attempt, and (3) the propensity scores only adjusted for observed confounder, not unobserved confounders. A strength of our study is that we had a national coverage of the studied outcomes and no loss to follow-up due to being based on register data.

## Conclusion

The high rates of suicide attempt and suicide at the time of discharge underscore the need for suicide preventive efforts. Also, six months after being discharged, high rates of suicidal behavior were found, which suggests that a support may be needed over a longer period of time after discharge than what was offered in the SAFE intervention. We found no effect on suicidal behavior of the SAFE intervention, and it is possible that this null finding can partly be explained by difficulties with implementing SAFE procedures fully in the intervention sites, and by the comparison sites implementing some of the SAFE procedures.

### Supplementary Information

Below is the link to the electronic supplementary material.

Supplementary file1 (DOCX 26 KB)

## Data Availability

The data that support the findings of this study are available from Statistics Denmark. Data access requires the completion of a detailed application form from the Danish Data Protection Agency, the Danish National Board of Health and Statistics Denmark. For more information on accessing the data, see https://www.dst.dk/en.
