# Supplementary material for: The effect of the SAFE intervention on post-discharge suicidal behavior: a quasi-experimental study using propensity score matching
Source: Soc Psychiatry Psychiatr Epidemiol. 2023 Nov 23;59(6):1053–61. doi: 10.1007/s00127-023-02585-y (PMC11116169; doi:10.1007/s00127-023-02585-y)
Supplement: Supplementary file 1 [file 127_2023_2585_MOESM1_ESM.docx]

eTable 1: Distribution of propensity score matched factors in discharges included in sensitivity analyses

	Shortened study period, i.e. September 1,2018 - March 31, 2020				Excluding all discharges where unipolar or bipolar disorder had been recorded as the main diagnosis				Followed until first suicide attempt			
	Safe site		Matched comparisons		Safe site		Matched comparisons		Safe site		Matched comparisons	
**Variables**	**n**	**%**	**n**	**%**	**n**	**%**	**n**	**%**	**n**	**%**	**n**	**%**
Sex												
Female	2741	47	2690	46	2600	43	2536	42	3256	46	3223	46
Male	3116	53	3167	54	3410	57	3474	58	3815	54	3848	54
Agegroups												
18-29 years	1594	27	1659	28	1717	29	1794	30	1809	26	1881	27
30-44 years	1801	31	1752	30	1943	32	1890	31	2230	32	2196	31
45-64 years	1858	32	1850	32	1872	31	1850	31	2290	32	2267	32
65+ years	604	10	596	10	478	8	476	8	736	10	727	10
Socio-economic status												
Working	1407	24	1559	27	1257	21	1324	22	1780	25	1886	27
Unemployed/social/sickness benef.	3395	58	3269	56	3771	63	3676	61	4021	57	3936	56
Retired	1012	17	991	17	934	16	959	16	1227	17	1208	17
Missing	43	1	38	1	48	1	51	1	43	1	41	1
Highest educational status												
Elementary school	2353	40	2500	43	2686	45	2758	46	2739	39	2899	41
Vocational training	1147	20	1133	19	1125	19	1168	19	1419	20	1440	20
Highschool	837	14	826	14	872	15	874	15	1061	15	1064	15
Bachelor degree or higher	1096	19	1055	18	875	15	841	14	1360	19	1264	18
Missing	424	7	343	6	452	8	369	6	492	7	404	6
Civil status												
Married	568	10	564	10	452	8	445	7	708	10	699	10
Divorced	969	17	959	16	940	16	893	15	1218	17	1143	16
Never married	4191	72	4211	72	4523	75	4571	76	4983	70	5047	71
Missing or other	129	0	123	0	95	2	101	2	162	2	182	3
Placed in foster care												
Yes	973	17	986	17	1156	19	1096	18	1128	16	1067	15
Primary diagnosis												
Substance misuse diag.	954	16	907	15	1239	21	1205	20	1130	16	1085	15
Schizophrenia spectrum diag.	2542	43	2454	42	3230	54	3195	53	3052	43	3023	43
Affective disorder	1205	21	1271	22	0	0	0	0	1507	21	1516	21
Anxiety or stress related disord.	552	9	580	10	732	12	779	13	693	10	733	10
Personality disorders	300	5	310	5	397	7	391	7	305	4	324	5
Admitted more than a week												
Yes	2741	47	2843	49	2492	41	2656	44	3379	48	3485	49
Ever comorbidity												
Substance misuse diagnoses	3589	61	3560	61	3882	65	3885	65	4154	59	4150	59
Schizophrenia spectrum diag.	3982	68	3908	67	4408	73	4395	73	4655	66	4571	65
Affective disorders	3388	58	3396	58	2725	45	2722	45	4001	57	4006	57
Previous suicide attempt												
Yes	2028	35	2028	35	2207	37	2207	37	2265	32	2265	32
Medical treatment last 12 months												
Anti-psychotic medicine (yes)	4756	81	4725	81	4710	78	4728	79	5538	78	5516	78
Anti-depressive medicine (yes)	4187	71	4187	71	3999	67	3999	67	4599	65	4599	65
Parent with history of psychiatric disorder												
Yes	1525	26	1472	25	1569	26	1521	25	1762	25	1640	23
Parent with history of suicidal behaviour												
Yes	482	8	444	8	511	9	497	8	581	8	510	7
